# Effectiveness of machine learning at modeling the relationship between Hi‐C data and copy number variation

**DOI:** 10.1002/qub2.52

**Published:** 2024-07-06

**Authors:** Yuyang Wang, Yu Sun, Zeyu Liu, Bijia Chen, Hebing Chen, Chao Ren, Xuanwei Lin, Pengzhen Hu, Peiheng Jia, Xiang Xu, Kang Xu, Ximeng Liu, Hao Li, Xiaochen Bo

**Affiliations:** ^1^ Institute of Health Service and Transfusion Medicine Beijing China; ^2^ College of Computer and Data Science Fuzhou University Fuzhou China; ^3^ Beijing Institute of Radiation Medicine Beijing China; ^4^ School of Life Sciences Northwestern Polytechnical University Xi’an China; ^5^ School of Mathematics and Computer Science Shanxi Normal University Taiyuan China; ^6^ School of Software Shandong University Qingdao China

**Keywords:** copy number variant, deep learning, graph convolution network, Hi‐C

## Abstract

Copy number variation (CNV) refers to the number of copies of a specific sequence in a genome and is a type of chromatin structural variation. The development of the Hi‐C technique has empowered research on the spatial structure of chromatins by capturing interactions between DNA fragments. We utilized machine‐learning methods including the linear transformation model and graph convolutional network (GCN) to detect CNV events from Hi‐C data and reveal how CNV is related to three‐dimensional interactions between genomic fragments in terms of the one‐dimensional read count signal and features of the chromatin structure. The experimental results demonstrated a specific linear relation between the Hi‐C read count and CNV for each chromosome that can be well qualified by the linear transformation model. In addition, the GCN‐based model could accurately extract features of the spatial structure from Hi‐C data and infer the corresponding CNV across different chromosomes in a cancer cell line. We performed a series of experiments including dimension reduction, transfer learning, and Hi‐C data perturbation to comprehensively evaluate the utility and robustness of the GCN‐based model. This work can provide a benchmark for using machine learning to infer CNV from Hi‐C data and serves as a necessary foundation for deeper understanding of the relationship between Hi‐C data and CNV.

## 1 INTRODUCTION

Copy number variation (CNV) refers to an increase or decrease in the number of copies of a DNA sequence in a genome, which can include duplications or deletions [1]. Previous works have revealed that human diseases accompany CNV events [2, 3], and they play an important role in hereditary illnesses [4]. Hi‐C sequencing is a high‐throughput method that uses restriction enzymes to detect the frequency of interactions between spatially adjacent genomic fragments and reveal their relative positions in three‐dimensional (3D) space, which can help with understanding the genomic structure [5, 6]. Hi‐C data encode CNV information due to the multiple copies of DNA fragments, which change the 3D spatial organization of the genome [7]. For instance, the observed number of sequencing reads corresponding to chromosomal interactions will be larger than expected in a region with an increased copy number [8]. CNV may subsequently be implicit in promoting aberrant gene expression patterns contributing to cancer initiation and progression. Thus, identifying CNV from Hi‐C data can help in determining their implications for higher‐order features of the chromatin structure, such as the formation of topologically associating domains and loops.

Several studies have attempted to predict CNV by analyzing Hi‐C data [9, 10, 11]. Hi‐C technology has become a powerful tool for capturing information on the interactions and spatial structures of chromatins [12]. Hi‐C data can be used to visually confirm changes in the 3D structure due to known genomic rearrangements and reveal the relationship between the spatial arrangement of a genome and its functions [13]. A CNV event can cause the higher read counts in a region than expected due to abnormally increased copies [9]. If CNV can be detected from Hi‐C data, this may help provide insight into how CNV affects the 3D interactions between genomic fragments involved in the expression levels of genes and regulatory factors. Various methods have been developed to detect CNV from the interaction signal in Hi‐C data [10, 14]. One approach has been to create a one‐dimensional (1D) coverage profile across the genome by calculating row or column sums of the interaction matrix and using the Hi‐C normalization method to remove the Hi‐C internal biases and improve the CNV prediction accuracy [9]. However, current computational methods are limited at qualifying the complex relationship between Hi‐C data and CNV because of the large data volume, data sparsity, and sequencing noise.

In recent years, machine learning has demonstrated its potential as a tool for modeling complex biological mechanisms and solving an increasingly diverse range of problems [15, 16]. Significant progress has been made in applying machine learning to large‐scale genome analysis and overcoming the sparsity and noise of genomic data. One of the primary applications has been Hi‐C map prediction, which involves predicting the pattern of chromosomal contacts between genomic sequences. Several deep learning‐based methods have been proposed for contact map prediction including DeepTACT [17], DeepC [18], and Akita [19], which have successfully extracted features from large‐scale DNA sequence data and generated corresponding Hi‐C maps. Such deep learning‐based methods have achieved a better performance than traditional methods, and deep learning has been applied to other tasks of Hi‐C data analysis as well, such as data enhancement. For instance, Zhang et al. proposed a deep learning approach to generate high‐resolution Hi‐C map data from low‐resolution Hi‐C data at a fraction of the normal sequencing cost while reducing noise in the Hi‐C data [20]. Another essential task in Hi‐C data analysis is the identification of chromosomal structural variation. Wang et al. developed a deep learning framework called EagleC that uses a convolutional neural network for detecting the full range of structural variation from Hi‐C data [21]. They achieved state‐of‐the‐art accuracy and sensitivity for structural variation detection compared to traditional methods. Overall, the application of machine learning to Hi‐C data analysis has resulted in significant improvements in accuracy and efficiency while providing insights into the 3D structure of the genome.

The identification of CNV from Hi‐C data is a challenge due to the sparsity of the Hi‐C data. To overcome this challenge, we employed various machine‐learning methods to establish a relationship between Hi‐C data and CNV based on the 1D interaction signal of each bin and the spatial information of chromosomes. We comprehensively evaluated the performance of these methods with the aim of obtaining insights into the strengths and limitations of different machine‐learning approaches at predicting CNV from Hi‐C data.

## 2 RESULTS

### 2.1 Study design

**FIGURE 1:**
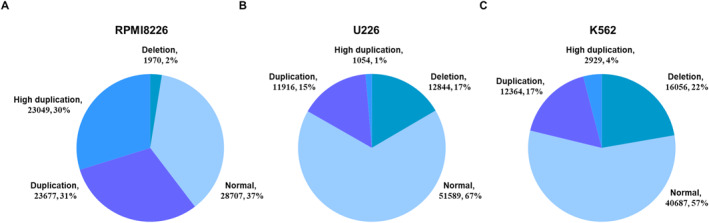
Distributions of standard CNV labels across cancer cell lines. We apply the Control‐FREEC to the WGS data of the RPMI8226, K562 and U226 cell lines and compute the copy number variations. (A), (B) and (C) indicate the numbers and distribution of the four CNV labels in the RPMI8226, U226 and K562 cell lines respectively. CNV, copy number variation; WGS, whole genome sequencing.

We collected Hi‐C data from three cancer cell lines: the chronic myelogenous leukemia cell line K562 and the two multiple myeloma cell lines U266 and RPMI8226. We calculated CNVs using the standard labels (i.e., 1: deletion, 2: normal, 3: duplication, 3+: high duplication) from the whole genome sequencing (WGS) of each cancer cell line using Control‐FREEC. We considered the computed CNVs as the ground truth for training the machine‐learning models. The label distributions are shown in Figure 1.

**FIGURE 2:**
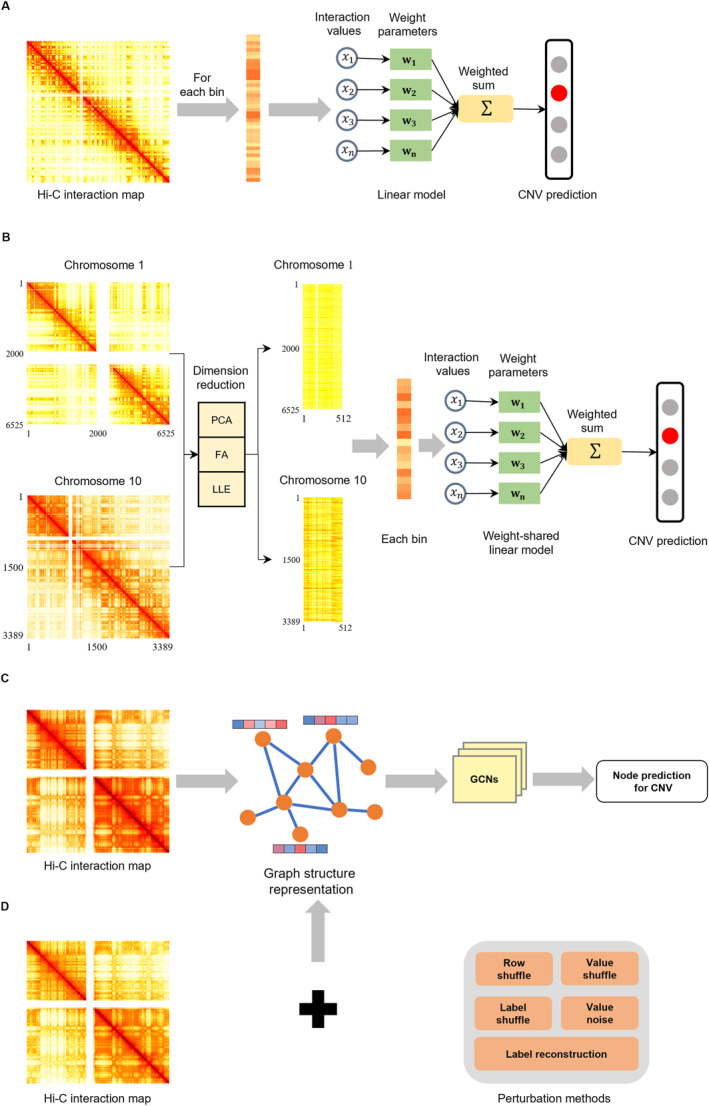
Overview of the study design. (A) Linear model to establish the relationship between the CNV and Hi‐C read count and construct specific regression coefficients for different chromosomes. (B) Dimensional reduction to unify dimensions of all chromosomes. Training and evaluation of the performance of a weight‐shared linear transformation model across different chromosomes. (C) Conversion of the Hi‐C interaction map into a graphical structure and application of the GCN‐based model to exploring the relationship between Hi‐C data and CNV. (D) Design of a series of perturbation experiments for Hi‐C data to evaluate the performance of the GCN‐based model. CNV, copy number variation; GCN, graph convolutional network.

First, we investigated how the number of DNA fragment copies affects the interaction strength between different genomic regions in the Hi‐C map. We applied the linear regression method to the 1D interaction signal of the Hi‐C map to quantify the relationship between the Hi‐C read count and CNV. Specifically, we constructed the linear model and applied it to a chromosome (Figure 2A). One weight‐shared linear model is applied to infer the CNV across the different chromosomes with the dimension reduction method (Figure 2B). While studying the relationship between CNVs and the chromatin structure can provide insights into understanding gene expression and regulation and genetic diseases, a linear transformation model of the 1D interaction signal in the Hi‐C map can hardly represent information on the spatial structure. A graphical structure is a powerful way to express complex relationships and offers significant advantages for representing network data. Accordingly, we considered the Hi‐C map as a graphical structure. Each node represented a bin in a chromosome, and the edges represented their interactions.

We applied a graph neural network (GNN) [22] for data analysis, which is a computational method for modeling the complex interactions and hierarchical relationships in a graphical structure. In genomic data analysis, GNNs can effectively represent the potential features of complex genes and transcription factors [23]. We trained a graph convolutional network (GCN)‐based model to effectively capture the features of the chromatin structure and predict the CNV of each bin on the Hi‐C interaction map while exploring the relationships between the Hi‐C data and CNV (Figure 2C) to understand the mechanism behind chromosome spatial interactions. We also performed a series of Hi‐C data perturbation experiments to evaluate the ability of the GCN‐based model to obtain critical features of the chromosomal structure related to CNV (Figure 2D).

### 2.2 Effectiveness of the linear transformation model

**FIGURE 3:**
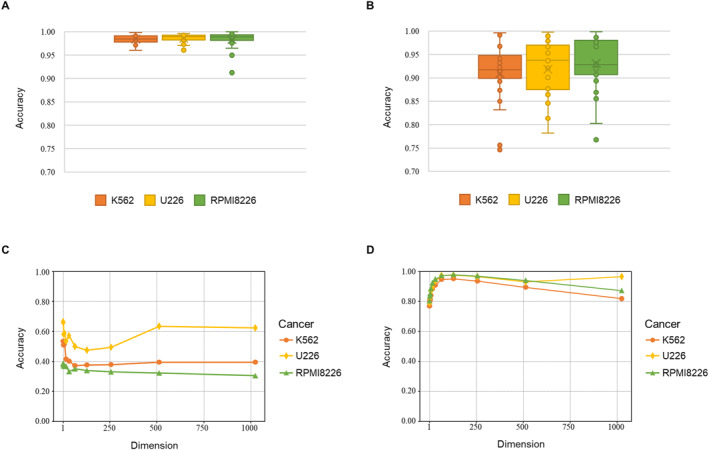
Results of the linear transformation model when applied to the Hi‐C data of the U226, RPMI8226, and K562 cell lines. (A) Prediction accuracy using 60% of the raw Hi‐C data for training and 40% for testing. (B) Prediction accuracy using 10% of the raw Hi‐C data for training. The detailed performance for each chromosome is represented as scatter points. (C) Prediction accuracy using one unified linear transformation model for all chromosomes and different dimensions. (D) Prediction accuracy using principal component analysis on 24 linear transformation models with different dimensions.

We first used the linear transformation model to determine the relationship between the chromatin interaction signal and CNV. The experimental datasets were from the K562, U226, and RPMI8226 cell lines and included Hi‐C data at 40‐kb resolution and CNV data. The dimensions of the linear transformation model depend on the chromosome length, which varies substantially. Therefore, we built an individual model for each chromosome with specific dimensions. We randomly selected 60% of the data for each chromosome for training and the other 40% for testing. The prediction accuracy is defined as the proportion of CNV bins that are correctly predicted among all chromosomes in a cell line. On average, the linear transformation model achieved prediction accuracies of 97.64%, 98.80%, and 98.67% for K562, U226, and RPMI8226, respectively (Figure 3A), and the prediction accuracy was above 96% for all chromosomes. The linear transformation model assumes the target variable (i.e., CNV) to be a linear combination of independent input variables (i.e., interaction signals), and the linear pattern between the two variables can be modeled by fitting a series of parameters that define the linear equation to the observed data. The results indicated a clear linear pattern between the Hi‐C read count and CNV for each individual chromosome, which is consistent with the established observation that the Hi‐C read count is linearly impacted by CNV events. We tested the generality of the linear transformation models by reducing the percentage of training data from 60% to 10% of the Hi‐C read count data and evaluated the CNV prediction accuracy. The linear transformation model still achieved an average prediction accuracy of 92% across the cell lines (Figure 3B) with decreases of 5.95%, 7.7%, and 5.45% for K562, U226, and RPMI8226, respectively. Only a few chromosomes showed a noticeable reduction in prediction accuracy. These results suggest that the linear relationships across different bins are nearly identical within a chromosome.

### 2.3 Similarity of linear patterns across different chromosomes

**FIGURE 4:**
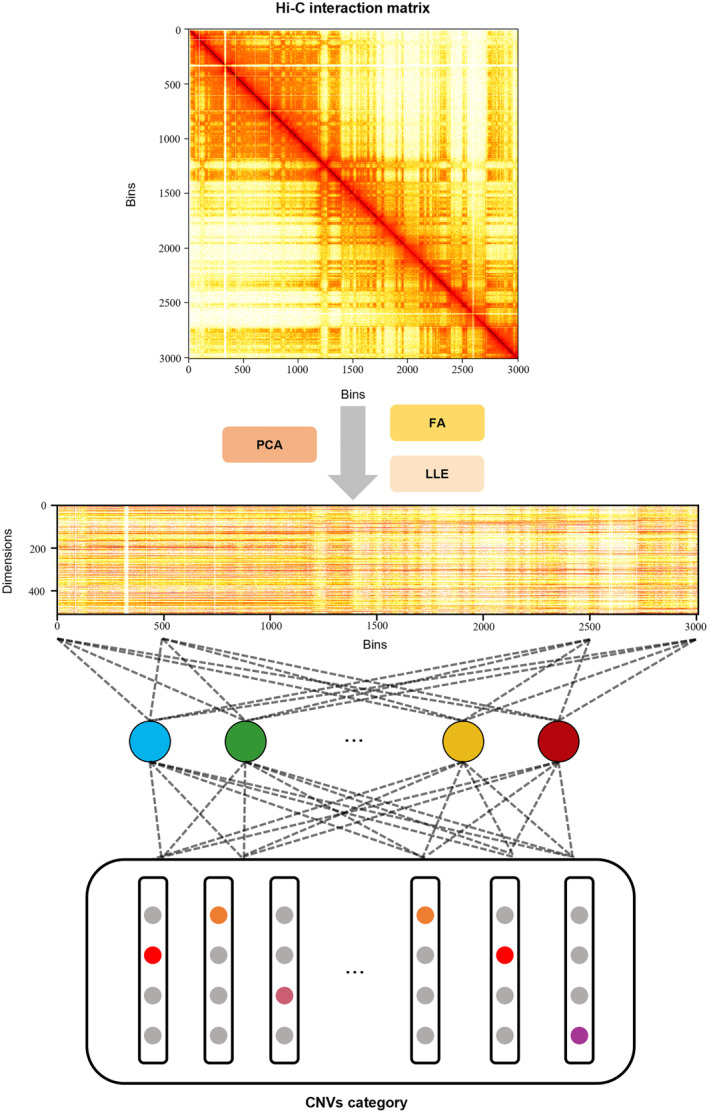
CNV prediction of the linear transformation model after dimensional reduction. We performed dimensional reduction on each chromosome in the cancer data to unify the dimensions. We utilized PCA, FA, and LLE to test their effectiveness. We then constructed a linear transformation model to predict the CNVs for each bin in a chromosome with the same dimensions. CNV, copy number variation; FA, factor analysis; LLE, locally linear embedding; PCA, principal component analysis.

Next, we investigated if an analogous linear correlation is present across different chromosomes. We attempted to train a weight‐shared linear transformation model to fit all chromosomes in a cell line and evaluate the CNV prediction accuracy. However, this could not be achieved directly because of the different dimensions. A practical solution was to perform dimensional reduction and unify the dimensions for the Hi‐C contact map of all chromosomes. This allowed the training of a weight‐shared linear transformation model rather than individual linear transformation models for each chromosome. The computational process is shown in Figure 4. We applied the same strategy of using 60% of the data for training and the remaining 40% for testing. We then evaluated the similarity of the linear patterns for different chromosomes in a cell line. Using a single unified linear transformation model for all chromosomes resulted in a significant reduction in the prediction accuracy (Figure 3C) compared to the performance of individual linear transformation models for different chromosomes (Figure 3A). Specifically, the weight‐shared linear transformation model obtained prediction accuracies of 48.79%, 34.28%, and 32.44% at 64 dimensions for U226, RPMI8226, and K562, respectively. The best prediction accuracies were 68.94%, 51.46%, and 39.83%, respectively. We then investigated the prediction accuracy after the dimensional reduction of the individual linear transformation models via principal component analysis (PCA). The individual linear transformation models achieved prediction accuracies of 95.20%, 97.64%, and 97.99% for K562, U226, and RPMI8226 at 64 dimensions (Figure 3D). This performance is similar to that in the previous experiment using raw Hi‐C data with the original dimensions. These results indicate that each chromosome has a particular linear pattern that cannot be generalized to the others and provide strong evidence for the previous claim that the linear patterns of different chromosomes do not hold for each other.

**FIGURE 5:**
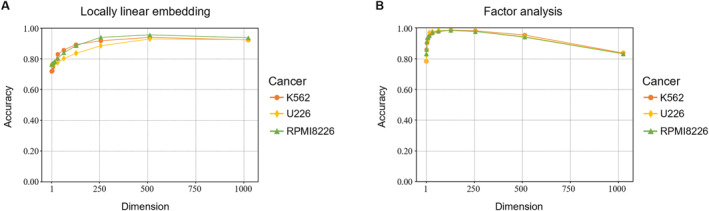
CNV prediction results using two different dimension reduction methods to the Hi‐C data of the cancer cell lines. (A) indicates the prediction accuracy using the locally linear embedding and (B) uses the factor analysis method to reduce the dimension of the raw Hi‐C data for CNV label prediction with the linear transformation model.

We investigated the effect of the dimensional reduction method used on the CNV prediction accuracy. We performed two additional experiments using different dimensional reduction methods to obtain the weight‐shared linear transformation model: locally linear embedding (LLE) and factor analysis (FA). The model obtained by LLE achieved prediction accuracies of 92.87%, 95.46%, and 93.81% for K562, U226, and RPMI8226, respectively (Figure 5A). The prediction accuracy was decreased compared to the model obtained by PCA. This demonstrates that a manifold learning‐based dimensional reduction method such as LLE is unsuitable for processing Hi‐C data for CNV prediction. In contrast, the model obtained by FA achieves prediction accuracies of 98.34%, 98.29%, and 98.57% for K562, U226, and RPMI8226, respectively (Figure 5B). FA is a linear reduction method like PCA and obtained the highest prediction accuracy among the dimensional reduction methods. FA can find critical information or elements from each bin, and it can be applied to Hi‐C data to find similar patterns across different chromosomes despite different cell lines. These results also suggest that CNV events can be detected precisely from a region of Hi‐C read counts. In other words, the linear transformation model can predict CNV only using a few of the critical Hi‐C read counts.

### 2.4 Utility of GCN for predicting CNVs from Hi‐C data

The above experiments demonstrated that the linear transformation model could identify the pattern between the Hi‐C read count and CNV within each chromosome using the 1D signal. However, the linear transformation model only considers interaction signals directly related to the current bin and overlooks global structural features. Here, we represented the Hi‐C interaction map as a graph and inferred CNV from the spatial structure of the chromosome by using the GCN‐based model. We used a weight‐shared GCN‐based model on all chromosomes to explore the relationship between the spatial structural features and CNV across different chromosomes. We trained the model on 60% of the graph nodes and tested the CNV prediction accuracy on the other 40% of the nodes. The GCN‐based model achieved an average prediction accuracy of 95.95% across the three cell lines (Table 1). The GCN‐based model demonstrated the power of the underlying convolutional module to extract potential features of the chromosomal structure and qualify the relationship between the chromosomal spatial structure and CNV. In addition, the weight‐shared GCN‐based model is naturally applicable to different chromosomes without preprocessing the Hi‐C data, in contrast to the linear transformation model. Notably, the weight‐shared GCN‐based model can accurately predict CNV across different chromosomes in a cell line. We concluded that the structural features associated with CNV have highly similar patterns across different chromosomes.

**TABLE 1:** Prediction performance of the GCN‐based model with the raw Hi‐C data.

Cell line	Accuracy (%)	*F*1 score	AUC
U226	94.87	0.9424	0.9689
RPMI8226	95.81	0.9553	0.9863
K562	97.18	0.9708	0.9829

Abbreviations: AUC, area under the receiver operating characteristic curve; GCN, graph convolutional network.

In addition, we tested the GCN‐based model on Hi‐C data processed by iterative correction and eigenvector decomposition (ICE) [24]. Compared to the raw Hi‐C data, ICE processing of the Hi‐C data decreased the average prediction accuracy of the model by 0.48% (Table 2). This indicates that ICE may cause a loss of information on the chromosomal structure. This will make it a challenge for the GCN‐based model to distinguish structural features from various CNV events, which can decrease the prediction accuracy.

**TABLE 2:** Prediction performance of the GCN‐based model with the Hi‐C data processed by ICE.

Cell line	Accuracy (%)	*F*1 score	AUC
U226	94.39	0.9430	0.9627
RPMI8226	94.65	0.9448	0.9518
K562	95.58	0.9542	0.9652

Abbreviations: AUC, area under the receiver operating characteristic curve; GCN, graph convolutional network; ICE, iterative correction and eigenvector decomposition.

We compared the GCN‐based model with the HiNT [9] and HiCnv [10], two mainstream methods that infer the CNV events from Hi‐C data mainly based on the Markov model. The results of the prediction performance are shown in Table 3. Our GCN‐based achieved the best performance on all three cell lines.

**TABLE 3:** Compare the prediction accuracy of the GCN‐based model with the HiNT and HiCnv on three cell lines. The GCN‐based model achieved the best performance in all cell lines.

Method	Cell line		
	U226	RPMI8226	K562
GCN‐based model	**94.87**	**95.81**	**97.18**
HiNT	83.18	80.42	86.75
HiCnv	70.04	66.43	68.12

*Note*: The bold values indicate the best prediction accuracy in all cell lines among different methods.

Abbreviation: GCN, graph convolutional network.

Then, we evaluated the GCN‐based model with a node feature of different dimensions on three cell lines. The results are shown in the Table 4, indicating that the GCN‐based model is capable to deal with different dimensions. Specifically, a lower dimension (e.g., 16) might moderately impair the performance of the GCN‐based model, while dimensions as high as over 32 would guarantee a satisfactory and stable performance.

**TABLE 4:** The prediction accuracy of GCN‐based with different bin node feature dimensions.

Cell line	Node feature dimension	Accuracy (%)
RPMI8226	16	88.79
	32	93.17
	64	**95.81**
	128	95.74
U226	16	91.45
	32	94.56
	64	**94.87**
	128	94.12
K562	16	89.26
	32	95.72
	64	97.18
	128	**97.83**

*Note*: The Node2vec algorithm is applied to obtain a node feature embedding for each bin and results in a series of node feature dimensions to explore the impact of GCN‐based model performance. The bold value is the best prediction accuracy in each cell line.

Abbreviation: GCN, graph convolutional network.

**FIGURE 6:**
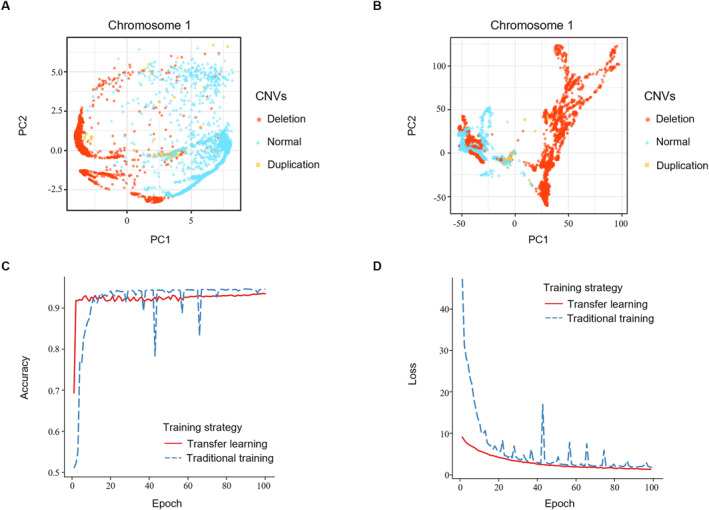
CNV prediction from Hi‐C data with the GCN‐based model. (A) Application of PCA to visualize the structural features embedded in chromosome 1 of the U226 cancer cell line with 64 dimensions obtained from the last convolutional layer of the GCN‐based model. (B) Chromosome clustering results for Hi‐C raw data of the U226 cancer cell line with PCA. (C) Comparison of the GCN‐based model performances with two different training strategies. (D) Training loss value curves of the GCN‐based model with two different training strategies. CNV, copy number variation; GCN, graph convolutional network; PCA, principal component analysis.

We clustered the structural features of chromosome 1 obtained by the GCN‐based model with 64 dimensions for each bin (Figure 6A). Compared to the clustering results obtained by applying PCA to the raw Hi‐C data (Figure 6B), the trained GCN‐based model was highly effective at capturing the structural features of chromosomal bins. Specifically, the model could distinguish between chromosomal structural features associated with different CNV events.

### 2.5 Transfer learning model

In another experiment, we applied the transfer learning strategy to evaluate the relationship between chromosomal structural features and CNV in an effort to develop a general method of identifying CNV across different chromosomes. We first trained the GCN‐based model on one cancer cell line (i.e., RPMI8226) and evaluated the performance on another cancer cell line (i.e., U226) without retraining. However, the prediction accuracy was only 69.31% with a large gap compared to the performance with a traditional training strategy (Figure 6C). This suggests that CNV has different associations with the chromosomal structure across different cancer cell lines. Notably, however, the GCN‐based model with the transfer learning strategy could accurately predict CNV by using 2% of the labeled cancer data. When we used 2% of the labeled U226 Hi‐C data to fine‐tune a GCN‐based model trained on 60% of the labeled RPMI8226 Hi‐C data, we achieved a prediction accuracy of 93.21%, which is similar to the performance achieved with the traditional training strategy (Figure 6C). These results indicate that using only a small amount of information on the chromosomal structure can adjust the association pattern between chromosomal structural features and CNV among different cell lines. The GCN‐based model also showed faster convergence with transfer learning than with the traditional training strategy by requiring no more than five epochs (Figure 6D). These findings suggest differences in the structural features of chromosomes between different cell lines. However, the differences can be corrected by a small amount of structural information to help the GCN‐based model precisely identify CNV.

### 2.6 Model performance with perturbed Hi‐C data

**FIGURE 7:**
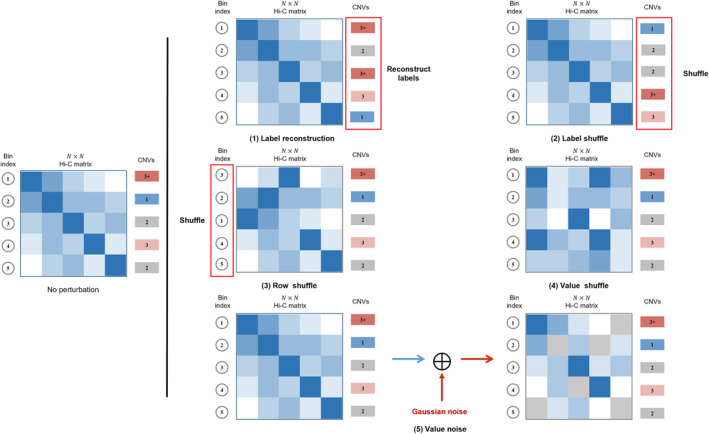
Different perturbation methods applied to the Hi‐C matrix data. Given a Hi‐C matrix with *N* bins, we designed five perturbation methods: label reconstruction, label shuffling, row shuffling, value shuffling, and value noise. For label reconstruction, we randomly reproduced CNV labels for each bin used to train and test the GCN‐based model. For label shuffling, we randomly exchanged labels among bins for a chromosome. For row shuffling, we exchange whole rows of bin values without exchanging the corresponding CNV labels. For value shuffling, we exchanged elements of the Hi‐C matrix for each chromosome without shuffling the row positions of the bins and CNV labels. For value noise, we added noise obeying a Gaussian distribution with the mean of 0 and variance of 1 to some of the elements in the Hi‐C matrix. CNV, copy number variation; GCN, graph convolutional network.

**FIGURE 8:**
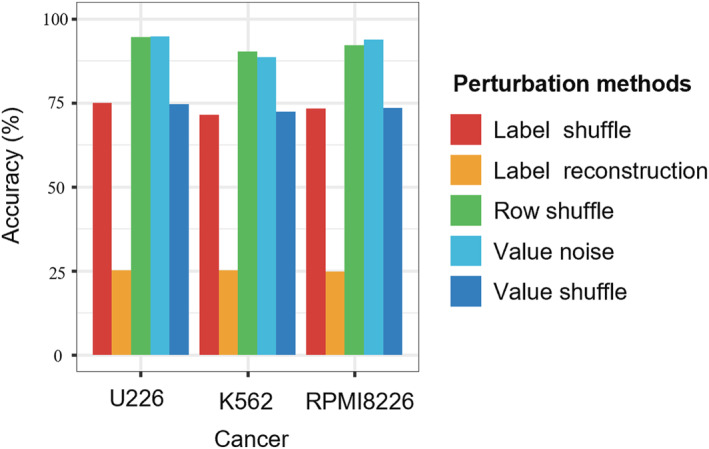
CNV prediction performance of the GCN‐based model across different cell lines with different perturbation methods. The Hi‐C data perturbation experiments result in a series of model performance changes. The model is robust for the row shuffle and value noise perturbation methods while sensitive to CNV label perturbation, including label reconstruction and shuffle methods. CNV, copy number variation; GCN, graph convolutional network.

Following the previous experiment, we introduced a series of perturbations to either the Hi‐C contact map or CNV labels to evaluate how closely the chromatin structure was associated with CNV (Figure 7). First, we perturbed the CNV labels by label reconstruction and shuffling and evaluated the performance of the GCN‐based model (Figure 8). For the label reconstruction, we randomly generated a new series of CNV labels for each chromosome. Then, we used 60% of the Hi‐C data to train the GCN‐based model and the other 40% for testing. The average accuracy of the GCN‐based model declined sharply to only 25.09% across the three cancer cell lines. As expected, label reconstruction made the model unable to match the original structural features related to CNV events to the correct CNV labels. Then, we shuffled the CNV labels for each chromosome and used the same training strategy. The GCN‐based model achieved prediction accuracies of 75.03%, 73.34%, and 71.52% for the U226, RPMI8226, and K562 cancer cell lines, respectively. However, we examined the distribution of CNV labels before and after shuffling and found that the genomic regions whose CNV label was unchanged comprised up to 61.31% of the total data. In other words, the label shuffling results do not accurately reflect the model performance in perturbed scenarios. We then randomly shuffled rows of a chromosome Hi‐C matrix and trained the GCN‐based model on 60% of the bins of Hi‐C data. This had a negligible effect on the performance of the GCN‐based model and reduced the prediction accuracy by only 0.17% compared to the traditional training strategy with the U226 Hi‐C data. We compared the original Hi‐C matrix with the shuffled matrix and found that 24% of the interaction values were unchanged. Importantly, we found that the perturbed graphical structure showed only slight changes due to the symmetry of the Hi‐C data. The GCN‐based model mainly focuses on the domain relationship of the current node. In other words, shuffling rows did not directly change the domain information, so the performance of the GCN‐based model was hardly affected. Finally, we perturbed random values of the Hi‐C in a fine‐grained manner while keeping the CNV labels of the chromosomal bins. For instance, we exchanged elements in a Hi‐C matrix, which decreased the prediction accuracy to 75.69% for U226. Exchanging elements destroyed the original chromosomal structure, which made the GCN‐based model unable to capture the structural features. In contrast, adding standard Gaussian noise to the elements of the Hi‐C interaction map had a negligible effect on the GCN‐based model, which still achieved a prediction accuracy of 94.70% for U226.

In summary, perturbing the CNV labels had an enormous influence on the prediction accuracy. This proves that the GCN‐based model is not driven by CNV during prediction and that it truly qualifies the relationship between chromosomal structural features and CNV. In addition, adding Gaussian noise reduced the average prediction accuracy by only 3.56%, which demonstrates the robustness of the GCN‐based model. To eliminate Hi‐C sequencing noise, mining information on structural variations from the structural characteristics of chromosomes can bring better results.

## 3 DISCUSSION

Here, we applied two effective machine‐learning methods to identify the CNV from Hi‐C data to overcome the challenge of the sparsity of the Hi‐C data. Through that, we first establish the relationship between Hi‐C data and CNV from the 1D interaction signal of each bin and the spatial information of chromosomes. Furthermore, we report comprehensive experimental results of proposed CNV inferred models. We discover that the linear transformation model could accurately predict CNV from the 1D signal of Hi‐C read counts, which indicates a strong linear relationship between the observed Hi‐C read counts and CNV. However, such a linear pattern varies from one chromosome to another, and it cannot be generalized to the whole genome, even for the same cancer. The GCN‐based model was able to identify structural features across different chromosomes in the same cancer. We utilized the transfer learning strategy to explore the differences in the chromatin structure between cancers. We validated the structural features learned by the GCN‐based model by examining the effect of perturbing the Hi‐C data and CNV labels on the model performance. The GCN‐based model demonstrated its robustness against noise from Hi‐C sequencing. In conclusion, we obtained experimental evidence demonstrating and quantifying the correlation between the Hi‐C contact map and CNV in terms of the 1D read count signal and 3D chromatin structure. The findings of this study may help guide subsequent research on the chromatin structure and CNV.

## 4 METHODS

### 4.1 Preparation of datasets

We directly downloaded the Hi‐C datasets of the RPMI8226 and U226 cancer cell lines from GEO (GSM2334834) and used the processed interaction matrices at a 40‐kb resolution that were mapped to the reference genome hg19. For the K562 cancer cell line, we downloaded the interaction matrices with a 10‐kb resolution from the GEO database (GSE63525) and transformed them into a 40‐kb resolution by using the HiC‐Pro (v3.1.0) Python package. We utilized ICE [24] to normalize the raw interaction matrices of the three cancer cell lines. We constructed CNV labels for training and evaluating the models by using Control‐FREEC (v11.6). We first downloaded the WGS datasets from the GEO database as the input and then called CNV labels by Control‐FREEC at a 40‐kb resolution with the reference genome hg19 to ensure consistency with the Hi‐C interaction matrices.

### 4.2 Linear transformation model

We use linear transformation to calculate the CNV for each bin from the observed number of Hi‐C sequencing read count data. In linear transformation, the dimension depends on the length of the chromosome, which varies substantially. Therefore, we built individual models for each chromosome with specific dimensions. Suppose a chromosomal Hi‐C interaction map matrix **X** = {**bin**_1_, **bin**_2_, …, **bin**_*n*_} containing *n* bins. Each bin is represented as a set of interaction values with other bins. The CNV of each bin can be calculated by the following linear transformation equation:

$$y={bin}_{i}w+b$$

where **bin**_***i***_ = {*x*_1_, *x*_2_, …, *x*_*n*_} is a vector that represents the *i*th row of the raw Hi‐C interaction matrix, **w** is a matrix of learnable weight parameters, and **b** is the corresponding bias vector for regularization. The multiplication of **bin**_***i***_ **w** can be considered as the process of summing the assigned weights for all interaction values **bin**_***i***_, which is a standard linear transformation method. Finally, **y** is a set of prediction probabilities of the CNV label for **bin**_***i***_. The CNV label with the highest probability is taken as the result.

### 4.3 Dimension reduction

The linear transformation model is limited at modeling the relationship across different chromosomes because of the various dimensions of Hi‐C read counts. We applied three dimension reduction methods to evaluate the performance of a unified linear transformation model across different chromosomes, PCA, FA, and LLE. For PCA, we first calculated the covariance matrix of each raw Hi‐C interaction matrix and computed the eigenvectors and eigenvalues of the covariance matrix. For dimensional reduction, we selected the number of eigenvectors corresponding to the highest eigenvalues equal to the target dimension. Notably, PCA assumes that the interaction data are linearly related and that the variables have a normal distribution. FA reduces dimensions by identifying the underlying factors that explain the covariance among observed variables. It mainly involves two stages. In the extraction stage, it first estimates the number of factors and then extracts these factors from the observed rows of Hi‐C read count data by using maximum likelihood estimation. In the rotation stage, orthogonal rotation is performed to force the extracted factors to be uncorrelated with each other, which simplifies the interpretation of each factor as representing a unique and independent dimension of the Hi‐C read count data. LLE is a nonlinear dimensional reduction technique that aims to project high‐dimensional data into a lower‐dimensional space while preserving the local structure of the data. For each data point of Hi‐C read counts, the k‐nearest neighbors are identified in the high‐dimensional data. A weighted linear combination of these neighbors is computed to reconstruct the original data point, which ensures that the local geometry of the data is preserved in the lower‐dimensional space. Then, global embedding is performed by using an optimization procedure to find a set of coordinates in the lower‐dimensional space that best approximates the original points while respecting the reconstructed weights. The above dimensional reduction methods were all implemented by using the scikit‐learn package in Python.

### 4.4 Pretraining the model for feature extraction

Hi‐C data can provide information on the spatial structure of a chromosome structure. We pre‐trained a node2vec model [25] to better represent the spatial structural features embedded in each bin. The first step was to represent the data as a graph. We considered the Hi‐C matrix as an undirected graph and built the corresponding chromosomal graph G ∈ {V, E}, where the node V is a bin in a chromosome and the weighted edge E represents the interactions between these nodes. Accordingly, the weight of an edge represents the strength of the interaction between two nodes. The node2vec algorithm was then trained on the graph. A weighted network was constructed from the input graph by assigning a weight to each edge based on its proximity to other nodes. The weights were then used to construct a biased random walk to explore the local and global structures of the graph. This generated a sequence of nodes that captured the structure of the graph. This sequence was used to train a skip‐gram model that learned the node embeddings. The skip‐gram model is a type of neural network that considers the context of a node to predict the target node. The model was trained to minimize the difference between the predicted and actual nodes in the context. This process was repeated several times until the embeddings converged. The obtained objective function for the node2vec model can be represented as follows:

$$\max_{f}\sum_{u\in V}\log\;P\left(N_{S}\left(u\right)|{\vec{w}}_{u}\right)$$

where ${\vec{w}}_{u}=f\left(u\right)\in R^{d}$ is the learned feature of node *u* ∈ V with *d* dimensions. We designed the feature embeddings to 64 features. The function $N_{S}\left(u\right)$ represents sampling the neighbor nodes of node *u*. We implemented and pretrained the node2vec model by using the node2vec package in Python. The initial hyperparameter walk length was set to 100, and the number of walks was set to 10. During the training stage, we set the window size to 10, which determined the number of nodes aggregated in a sample for training the model. Once the node2vec model was trained, we could obtain the input node features of each bin for the GCN‐based model to infer CNV events.

### 4.5 GCN‐based model

**FIGURE 9:**
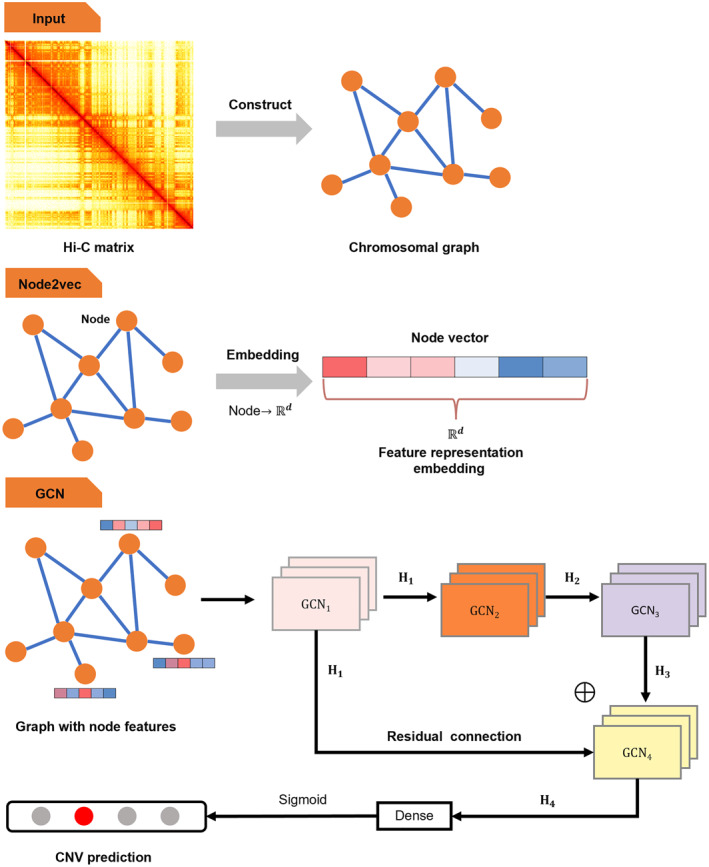
Architecture of the GCN‐based model comprising the input, node2vec, and GCN. A Hi‐C matrix with *N* bins is the input, and a chromosomal graph is constructed that treats the bins as the nodes. The interaction values between bins are considered weighted edges. The chromosomal graph is passed into the node2vec module, which learns feature representation for each node and represents it as a *d* node vector according to the structural information of the graph. Finally, the GCN module has four GCN layers that uses the chromosomal graph with node features as the input. The GCN layers recursively compute the hidden features of all nodes. Residual connection is used to prevent gradient vanishing. A dense layer with the sigmoid probability function is used to predict the CNV of each bin. CNV, copy number variation; GCN, graph convolutional network.

The GCN‐based model involved training a deep neural network to predict the CNV for each bin in a chromosome from a graphical representation of Hi‐C data. Figure 9 shows the process of the GCN‐based model. The input was the preprocessed Hi‐C matrix, which was represented as a chromosomal graph. We used the pretrained node embedding model node2vec and obtained all node representation vectors for the combined input of the GCN module. For the GCN module, we used four GCN layers to infer CNV events. We combined the graph structure information G and node features $\vec{W}$ from the node2vec module as the input. We then passed the input to the first GCN layer with the Tanh active function and obtained the hidden feature vector H_1_ for all nodes in the graph:

$$H_{1}=\mathrm{Tanh}\;\left(\text{GC}N_{1}\;\left(G,\vec{W}\right)\right)$$

After that, we then recursively used the hidden feature **H**_1_ as the node feature input for the following three GCN layers:

$$H_{2}=\mathrm{Tanh}\;\left(\text{GC}N_{2}\;\left(G,H_{1}\right)\right)$$

$$H_{3}=\mathrm{Tanh}\;\left(\text{GC}N_{2}\;\left(G,H_{2}\right)\right)$$

$$H_{4}=\mathrm{Tanh}\;\left(\text{GC}N_{4}\;\left(G,H_{1}+H_{3}\right)\right)$$

Notably, we used a residual connection to concatenate the first GCN layer’s output **H**_1_ and the third GCN layer’s output **H**_3_ as an input of the last GCN layer $\text{GC}N_{4}\;\left(∙\right)$. The residual connection method [26] prevents the problem of vanishing and exploding gradients in a deep neural network and promotes convergence. It can also relieve over‐smoothing in a multilayer GCN‐based model. Finally, we used a fully connected network layer with the dropout regularization method to prevent overfitting. We used the sigmoid active function to predict the probability of every CNV label for each node:

$$O=\text{Sigmoid}\;\left(\text{Dense}\;\left(H_{4}\right)\right)$$

To train the model, we constructed a training dataset by randomly choosing 60% of bins (nodes) in a chromosomal graph and computing the CNV labels **y** with Control‐FREEC by using the ground‐truth CNVs for each bin. We utilized one‐hot encoding for the CNV labels. Then, the model was trained by minimizing the following objective function:

$$\text{Loss}=\frac{1}{N}\sum_{i=1}^{N}\text{CrossEntropy}\;\left(O_{i},y_{i}\right)$$

We used the cross‐entropy as the loss function (objective function), and the *N* was the number of bins. We used the Adam optimizer to update the model parameters.

### 4.6 Perturbation methods

We utilized the series of perturbation methods to the Hi‐C data to evaluate the performance of the GCN‐based model. Suppose a *N* × *N* Hi‐C matrix where *N* is the number of bins. For label reconstruction, we used the random function in Python to generate a list of CNV labels. Specifically, we used the random function to sample a value from the {0, 1, 2, 3} collection as the reconstructed CNV label for each bin. We then used the original Hi‐C matrix with *N* reconstructed labels to train and test the GCN‐based model. For label shuffling, we applied the random.shuffle() function in Python to the original CNV label list of each chromosome and obtained a randomly sorted CNV label sequence. We did not change the values of the original label list. Then, we perturbed the original Hi‐C matrix. For row shuffling, we used the function random.shuffle() in Python on the original row index list and rearranged the Hi‐C matrix according to the shuffled list. For value shuffling, we exchanged all values in a chromosome Hi‐C matrix. We flattened the Hi‐C matrix into a one‐dimensional list and then used the random.shuffle() function to shuffle all values of the list. We then reshaped the list into a *N* × *N* Hi‐C matrix. For value noise, we added Gaussian noise to the values in the Hi‐C matrix. We applied the *numpy*.*random*.*normal* function to generate a *N* × *N* Gaussian matrix subject to a normal distribution (0 mean and 1 variance). We utilized the matrix to add Gaussian noise to the original Hi‐C matrix.

## AUTHOR CONTRIBUTIONS

**Yuyang Wang**: Formal analysis; methodology. **Zeyu Liu**: Formal analysis; methodology; validation. **Yu Sun**: Conceptualization; methodology; writing – original draft. **Bijia Chen**: Methodology; writing – original draft. **Hebing Chen**: Conceptualization; writing – review & editing. **Chao Ren**: Data curation; validation. **Xuanwei Lin**: Data curation. **Pengzhen Hu**: Data curation. **Peiheng Jia**: Investigation. **Xiang Xu**: Investigation. **Kang Xu**: Data curation. **Ximeng Liu**: Conceptualization; writing – review & editing. **Hao Li**: Conceptualization; methodology; writing – review & editing. **Xiaochen Bo**: Conceptualization; methodology; project administration; writing – review & editing.

## CONFLICT OF INTEREST STATEMENT

The authors Yuyang Wang, Yu Sun, Zeyu Liu, Bijia Chen, Hebing Chen, Chao Ren, Xuanwei Lin, Pengzhen Hu, Peiheng Jia, Xiang Xu, Kang Xu, Ximeng Liu, Hao Li and Xiaochen Bo declare that they have no conflict of interest or financial conflicts to disclose.

## ETHICS STATEMENT

This article does not contain any studies with human or animal materials performed by any of the authors.
